# Multibranch semantic image segmentation model based on edge optimization and category perception

**DOI:** 10.1371/journal.pone.0315621

**Published:** 2024-12-19

**Authors:** Zhuolin Yang, Zhen Cao, Jianfang Cao, Zhiqiang Chen, Cunhe Peng

**Affiliations:** 1 Department of Computer Science and Technology, Xinzhou Normal University, Xinzhou, China; 2 School of Computer Science and Technology, Taiyuan University of Science and Technology, Taiyuan, China; 3 Department of Big Data and Intelligent Engineering, Shanxi Institute of Technology, Yangquan, China; Purdue University, UNITED STATES OF AMERICA

## Abstract

In semantic image segmentation tasks, most methods fail to fully use the characteristics of different scales and levels but rather directly perform upsampling. This may cause some effective information to be mistaken for redundant information and discarded, which in turn causes object segmentation confusion. As a convolutional layer deepens, the loss of spatial detail information makes the segmentation effect achieved at the object boundary insufficiently accurate. To address the above problems, we propose an edge optimization and category-aware multibranch semantic segmentation network (ECMNet). First, an attention-guided multibranch fusion backbone network is used to connect features with different resolutions in parallel and perform multiscale information interaction to reduce the loss of spatial detail information. Second, a category perception module is used to learn category feature representations and guide the pixel classification process through an attention mechanism to optimize the resulting segmentation accuracy. Finally, an edge optimization module is used to integrate the edge features into the middle and the deep supervision layers of the network through an adaptive algorithm to enhance its ability to express edge features and optimize the edge segmentation effect. The experimental results show that the MIoU value reaches 79.2% on the Cityspaces dataset and 79.6% on the CamVid dataset, that the number of parameters is significantly lower than those of other models, and that the proposed method can effectively achieve improved semantic image segmentation performance and solve the partial category segmentation confusion problem, giving it certain application prospects.

## Introduction

Semantic image segmentation is a hot topic in the field of computer vision. Its purpose is to predict the categories of all the pixels in an input image. Semantic segmentation has great application value in many aspects of society, such as unmanned driving, intelligent medical research, robot navigation, and geographic information systems. In these practical applications, high-precision segmentation results are particularly important.

With the advancement of deep learning technology, pixel-level semantic segmentation faces several challenges, such as the difficulty of distinguishing objects with similar appearances or small sizes and the vulnerability of segmentation results to the external environment. To address these challenges, this paper proposes an image segmentation method based on boundary perception optimization and semantic spatial information feature fusion.

The main work of this paper is as follows.

1. An attention-guided multibranch fusion backbone network (AMFM) is used as the feature extraction module of this paper. It aims to maintain branch features with four different resolutions, thereby reducing the loss of spatial and detail information and enhancing the ability of the model to perceive fine-grained features. A bidirectional detail enhancement module (BDEM) is responsible for fusing spatial detail information and contextual semantic information, whereas an attention perception aggregation module (APAM) is used to fuse branches with different resolutions to enhance the semantic information contained in the high-resolution branches and optimize the overall feature representations.
2. A category awareness module (CAM) is designed to learn the feature representation of each category and capture the feature differences between categories. Moreover, multiscale context information is fully utilized to integrate the category feature information into the pixel feature representation, which provides context-dependent guidance for the pixels, improves the accuracy of semantic segmentation, and mitigates the confusion of object category segmentation.
3. The training process is supervised by an edge optimization module and a joint loss function based on edge computing. This paper proposes an improved adaptive Canny algorithm to enhance the spatial detail information of the network for each depth feature. During the training process, the network is designed to pay more attention to the edge of the target object to help generate more refined and accurate segmentation results, thus solving the problem of fuzzy object edge segmentation results produced by the network.

The main structure of the following sections is as follows. In the Related work section, we perform a literature review on articles with relevant topics and point out their drawbacks as well as the current literature gap that remains to be resolved. In the Methods section, we present the theoretical background related to the model proposed in this paper, detail the improvements it encompasses, and describe the experimental procedures. In the Results and discussion section, we analyze the segmentation results of our model by comparing them with those of other models, as well as presenting the results of the ablation experiments. The Conclusion section comes last.

## Related work

The traditional semantic image segmentation methods use low-level features such as grayness, spatial texture, color information and geometric shape features to segment an image into different parts. These methods include threshold-based segmentation methods [1], which distinguish the target from the background by comparing the gray values of pixels; the edge segmentation method [2], which combines salient object extraction and instance segmentation techniques, such as MASK R-CNN; and segmentation methods, which are based on texture (color) information or graph theory [3], where pixels are regarded as graph vertices and edges represent the relationships between pixels. Although these methods have fast segmentation speeds, they need to artificially design feature extractors, and their segmentation effects produced for complex scenes are not good. In recent years, deep convolutional neural networks (DCNNs) [4] have made breakthroughs in semantic segmentation tasks. The fully convolutional network (FCN) proposed by Long et al. [5] in 2015 extends the classification network from the image level to the pixel level and realizes end-to-end semantic segmentation.

Subsequent advancements related to pixel-level semantic segmentation are as follows. Miangoleh et al. [6] proposed a method for improving the quality of segmentation results by integrating multiresolution and multilevel features. Pu et al. [7] used complete image context information and detailed local clues to optimize the processes of extracting object boundaries and generating meaningful edges and ultimately achieved improved semantic image segmentation performance. The method of integrating multiresolution and multilevel features aims to fuse contextual semantic information to determine the correlations between pixels while retaining the spatial detail information of the input image. In semantic image segmentation, the preservation of spatial information is crucial for providing accurate predictions. The traditional continuous downsampling process often loses the spatial location and boundary details of the original image when obtaining deep features. SPMNet [8] introduces residual connections to enhance spatial relationships, and SCA-Net [9] uses a spatial attention block (SAB) in combination with multiscale information to obtain spatial features. Semantic context information is an important basis for judging the correlations between pixels, and it is also necessary for achieving high-quality segmentation effects. The experimental results produced by ENCNet [10], SoENet [11] and CFPNet [12] showed that context information is conducive to high-quality semantic segmentation. DenseASPP [13] concatenates multiscale features to obtain context information. RELAXNet [14] uses an efficient asymmetric bottleneck residual (EABR) to extract multiscale features. PCANet [15] designs a pyramid atrous attention module (PAA) to capture context information. The bilateral attention network (BANet) [16] uses a channel correlation coefficient to pay attention to the positive and negative dependencies between module-learned channel mappings and uses the weights of all channel mappings to update each channel mapping. The use of a bidirectional attention module can effectively integrate local and global information and eliminate ambiguity.

However, most of the above methods usually simply integrate multiscale feature information and fail to effectively address the representation differences between features with different scales, thus limiting the propagation effect of feature information in the utilized model. This situation causes some effective feature information to be mistaken for redundant information and discarded, which reduces the accuracy and sensitivity of segmentation for small categories and similar categories. Moreover, the existing methods do not consider the spatial distance between a pixel and the target boundary and cannot effectively measure local spatial changes.

## Methods

### Theoretical background

#### Multilayer features

In image segmentation tasks, low-level features have higher resolutions and more boundary information but lack rich semantic information and may contain more interference information. In contrast, high-level features have rich semantic information but low resolutions. To effectively aggregate different levels of feature information, many feature fusion modules have been proposed according to the existing image segmentation tasks. Zhao et al. [17] proposed a feature pyramid pooling module to fuse different levels of feature information, which improved the ability of their model to use global context information, but its segmentation effects for some small targets or edge details are still lacking. Chen et al. [18] used different dilated convolutions in an atrous spatial pyramid pooling (ASPP) module to effectively capture multiscale contextual information to improve the ability of their model to recognize targets at different scales. However, dilated convolutions with high sampling rates also cause the loss of spatial detail feature information. The context-aware cross-level fusion network (C2FNet) [19] uses an attention-induced cross-level fusion module (ACFM) to fuse different levels of feature information. A dual-branch global context module (DGCM) is used to fuse the rich global context information contained in cross-level features. The use and fusion of features at multiple different scales can combine information derived from different scales to better understand and describe the global and local features of objects. Doing so not only improves the robustness of the associated image segmentation model but also improves the detail processing ability and overall performance of the model. This strategy is widely used in various visual tasks.

#### Attention mechanisms

An attention mechanism can effectively capture the long-range correlations between different positions (pixels), and the weighted sum of all positions can be used to obtain the features of each position. Therefore, each position (pixel) can obtain a global view without a feature mapping reduction (resolution reduction). To date, various attention mechanisms in computer vision have been utilized, such as channel attention, spatial attention, temporal attention, branch attention, and their combinations [20]. The convolutional block attention module (CBAM) [21] uses an attention mechanism in both the spatial and channel dimensions to more effectively capture and utilize important features and improve the feature expression ability of the applied model. The attention network (ATTNet) [22] is a spatial bar attention module that uses 1×N and N×1 bar pooling kernels to extract features and avoids the irrelevant information and additional parameter calculations caused by traditional N×N pooling kernels to effectively capture the long-distance dependencies of local regions. The attention-aware fully convolutional context attention network (CANet) [23] uses an empty spatial pyramid attention module, which embeds a pixel similarity-based attention module in an empty spatial pyramid to enhance the connections between pixels and solve the pixel loss problem. The self-attention based neural network model (Segment Anything Model, SAM) calculates the attention weight of each position with respect to other positions and thus obtains a weighted representation of this position, which enhances the relationships between different positions in each layer. This model does not only exhibit strong robustness but realize precise segmentation in terms of object edges and details [24]. The residual-efficient learning and attention-expected fusion network (RELAXNet) [25] incorporates an attention mechanism into the jump connection between the encoder and decoder to promote the reasonable fusion of high-level features and low-level features. The Reviving Iterative Training with Mask Guidance (RITM) model [26] significantly improves the efficiency and effectiveness of interactive image segmentation by combining iterative training and mask guidance. Interactive image segmentation achieves the same goal as attention mechanisms by manually segmenting regions of interest and correcting segmentation prediction errors, and thus has important applications. The transformer architecture for optical flow estimation (TransFlow) utilizes spatial self-attention, cross-attention mechanisms and temporal correlations to achieve efficient global matching [27].

#### Edge enhancement

As an effective improvement method, edge enhancement technology can make a segmentation algorithm able to more accurately identify and distinguish the boundaries of different objects by highlighting the edge information contained in the input image, thus improving the accuracy and robustness of segmentation. Edge enhancement not only helps improve the ability of a model to capture details but also significantly improves its segmentation effect in complex scenes those with noise interference. The edge-enhanced multi-exposure fusion network (EEMEFN) introduces an edge enhancement module that refines the initial image using edge information, which enables the network to generate clear edge maps in low-light images [28]. The multi-level fusion based deep convolutional network for image quality assessment (MF-IQA) employs multi-level fusion and edge feature integration strategies to produce accurate and meaningful visual perception predictions [29]. Chen et al. [30] proposed an adaptive edge feature module in a neural network to enhance its ability to express edge features and the segmentation effect achieved for small objects. Moreover, the acquired edge information is fused with the feature information of the backbone network, and the result is used as the input of an auxiliary loss function for deep supervision to make the network pay more attention to the edge part. The latest boundary attention model proposed by Google [31] effectively alleviates the difficulty of capturing and understanding the key features of images taken in low-resolution scenes. By introducing intersection space parameterization to iteratively optimize the field, this model gradually refines the local boundary around each pixel to overcome the information loss challenge in low-resolution images. The STDC model proposed by Fan et al. [32] also integrates a detail guidance module into the network. Soft detail features with different sizes are generated by a 2D Laplacian operator and then upsampled and fused. The threshold is transformed into a binary feature map with edge information and used for supervised training, which improves the accuracy of the segmentation task. The use of edge enhancement technology in image segmentation tasks can highlight the edge information contained in an image, improve the ability of the employed segmentation algorithm to recognize the object boundary, and improve the accuracy and effect of segmentation.

### Method of this paper

In semantic segmentation tasks, multiscale feature information plays a decisive role in the prediction of semantic regions of different target objects, and accurate edge location and retention processes are crucial for obtaining fine segmentation results. Most of the existing semantic segmentation models focus on deep contextual relationships and fail to pay attention to details and edge information, so these models have weak abilities to learn the details and edge information of different semantic targets. To address this problem, this paper proposes an edge optimization and category-aware multibranch semantic segmentation network (ECMNet), which strengthens the process of learning edge and spatial details in the feature learning stage of the model.

**Fig 1:**
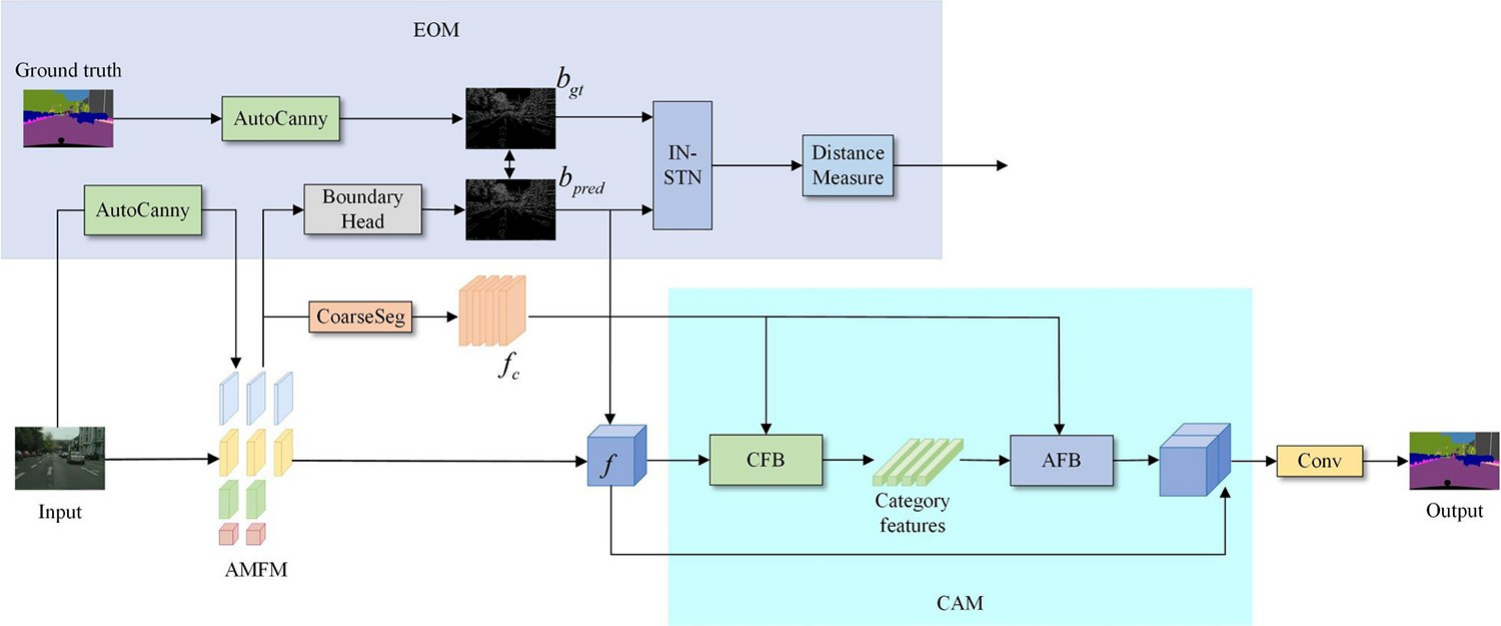
Multibranch network structure based on edge optimization and category awareness. AMFM, attention-guided multibranch fusion backbone module; EOM, edge optimization module; CAM, category-aware module; AFB, attention feature block; CFB, category feature block.

The overall structure of the network is shown in Fig 1. First, an attention-guided multibranch fusion backbone module (AMFM) is used to extract edge contours with the improved adaptive Canny algorithm (AutoCanny) to increase the attention given to the edge information. A bidirectional detail enhancement module (BDEM) is used to promote the interaction of feature information between branches with different resolutions to further focus on spatial details. Moreover, an attention perception aggregation module (APAM) is proposed, which can capture local spatial dependencies in parallel while maintaining global context relationships. Second, to emphasize the feature representation differences between categories and enhance the overall segmentation accuracy, a category-aware module (CAM) is designed to learn the feature representation of each category and use it to guide the classification decision of each pixel. Finally, given that segmentation boundaries are blurred in semantic segmentation tasks and that the commonly used pixel-level cross-entropy loss function is insensitive to edges and details, which makes it difficult to obtain clear boundaries, this paper proposes a joint loss function based on boundary-aware optimization, which enhances the deep supervision of the network in the training phase. The addition of an edge optimization module (EOM) optimizes the ability of the network to learn the edge information of different semantic targets without increasing the size and computational complexity of the segmentation network.

#### Multibranch backbone network

**Fig 2:**
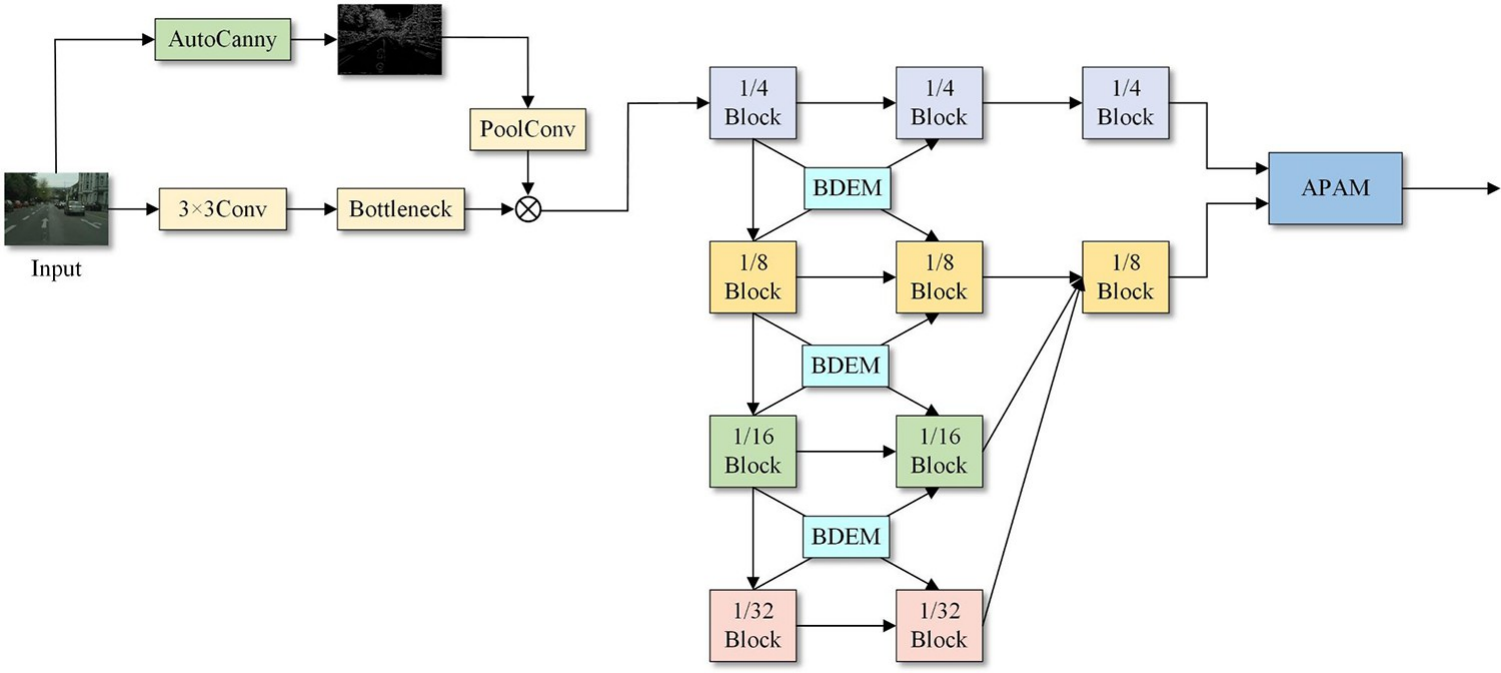
Attention-guided multibranch fusion backbone network. BDEM, bidirectional detail enhancement module. APAM, attention perception aggregation module.

This study proposes an AMFM inspired by the HRNet model, and its structure is shown in Fig 2. Considering the large number of calculations required for high-resolution input images, the four feature branches of the backbone network are set to 1/4, 1/8, 1/16 and 1/32 of the original image resolution. First, the input image is preprocessed and downsampled to 1/4 of its initial size, and the improved adaptive Canny edge detection algorithm is used to extract effective edge contour information and integrate it into the backbone network. Second, the preprocessed features are branched, and these branches correspond to the four blocks with different resolutions in the figure. A bidirectional detail enhancement module (BDEM) is used between each pair of adjacent resolution branches to promote full interaction between different branches and different levels of features and ultimately reduce the spatial and detail information losses. Finally, the attention perception aggregation module (APAM) is applied to the interactive features, which helps to alleviate the problem that spatial detail information is easily ignored during the feature fusion process.

(1) Bidirectional detail enhancement module (BDEM)

The bidirectional detail enhancement module (BDEM) supplements missing low-level detail features through bidirectional interaction between shallow spatial detail features and deep contextual semantic features. It suppresses redundant detail information while using an attention mechanism during feature interaction to improve the overall ability of the network to learn both detail and semantic information.

**Fig 3:**
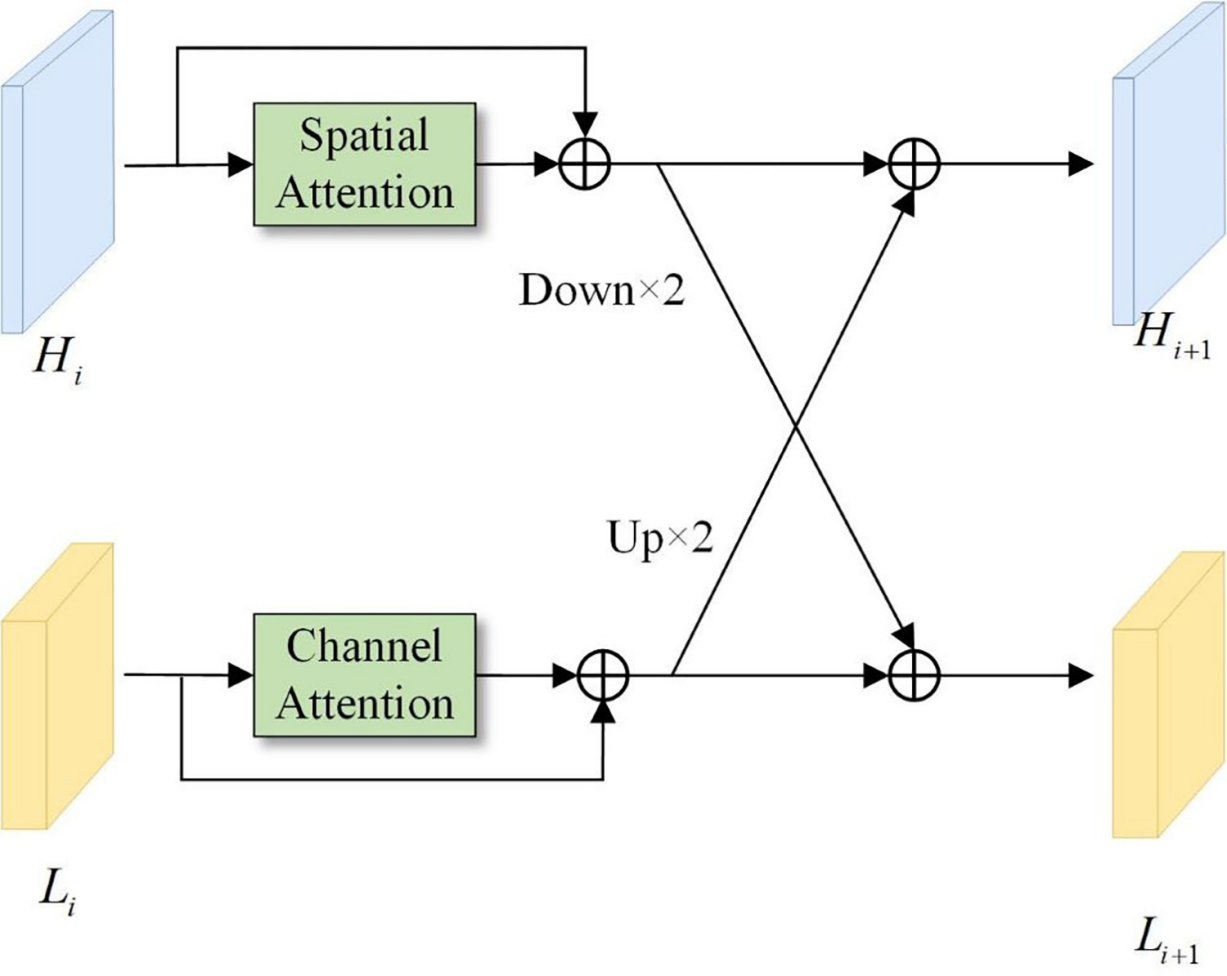
The structure of the bidirectional detail enhancement module (BDEM).

The associated process is as follows (Fig 3). First, the spatial detail features output by the high-resolution branch first obtain the corresponding spatial feature weights through a spatial attention calculation, and then the weighted spatial detail features are residually connected with the original spatial detail features to generate a high-resolution feature map after implementing detail enhancement. The feature map is then downsampled via a 3×3 convolution with a stride of 2, and it is added to the enhanced low-resolution semantic feature map. This process achieves the goal of injecting enhanced spatial detail features into low-resolution semantic features, which can be seen in Eq (1).

Second, the semantic feature map output by the low-resolution branch first obtains the corresponding weight through a channel attention calculation and then residually connects the weighted feature map with the original semantic feature map to obtain an enhanced low-resolution feature map. Then, the feature map is upsampled to a higher resolution via bilinear interpolation, and the elements are added to the high-resolution feature map, which is enhanced in terms of spatial details. This process achieves the goal of injecting deep contextual semantic features into high-resolution spatial detail features, which can be expressed via Eq (2).

$$L_{i+1}=(\mathrm{CA}(L_{i})+L_{i})+\mathrm{Down}(\mathrm{SA}(H_{i})+H_{i})$$ (1)

$$H_{i+1}=(\mathrm{SA}(H_{i})+H_{i})+\mathrm{Up}(\mathrm{CA}(L_{i})+L_{i})$$ (2)

where *L*_*i*_ and *L*_*i*+1_ represent the low-resolution semantic features observed before and after the spatial detail injection process, respectively. *H*_*i*_ and *H*_*i*+1_ represent the high-resolution detail features obtained before and after injecting contextual semantic features, respectively. CA (∙) represents the channel attention operation, and SA(∙) represents the spatial attention operation. Down(∙) represents the downsampling operation, which is implemented by a 3×3 convolution operation with a stride of 2. Up(∙) represents the upsampling operation, which is implemented via bilinear interpolation.

The spatial attention operation used in the BDEM module can be expressed as shown in Eqs (3) and (4), and the channel attention operation is implemented via Eqs (5) and (6).

$$H_{weight}=\mathrm{Sigmoid}(\mathrm{Conv}(\mathrm{Concat}(\mathrm{MaxPool}(H_{i}),\mathrm{AvgPool}(H_{i}))))$$ (3)

$$H_{e}=H_{i}+H_{i}\times H_{weight}$$ (4)

$$L_{weight}=\mathrm{Sigmoid}(\mathrm{MLP}(\mathrm{AvgPool}(L_{i}))+\mathrm{MLP}(\mathrm{MaxPool}(L_{i})))$$ (5)

$$L_{e}=L_{i}+L_{i}\times L_{weight}$$ (6)

where *H*_*weight*_ and *L*_*weight*_ calculate the spatial and channel attention weights, respectively, for the module and *H*_*e*_ and *L*_*e*_ are the enhanced high- and low-resolution branch feature maps output by the module. The spatial attention process first performs maximum pooling Max(∙) and average pooling AvgPool(∙) operations on *H*_*i*_ in the channel dimension, and the obtained single-channel feature map is spliced with the Concat(∙) operation. After convolution Conv(∙) processing, the Sigmoid(∙) activation function is used to obtain the weight *H*_*weight*_. Finally, the weight *H*_*weight*_ is multiplied by the corresponding element *H*_*i*_, and then the element is added to obtain an enhanced high-resolution branch feature *H*_*e*_. The channel attention mechanism sends the maximum pooling and average pooling results to a multilayer perceptron MLP(∙), the two 1×1×C feature maps output by the MLP are added and reactivated in a channel-by-channel manner, and finally, the channel attention weight *L*_*weight*_ is obtained.

(2) Attention perception aggregation module (APAM)

**Fig 4:**
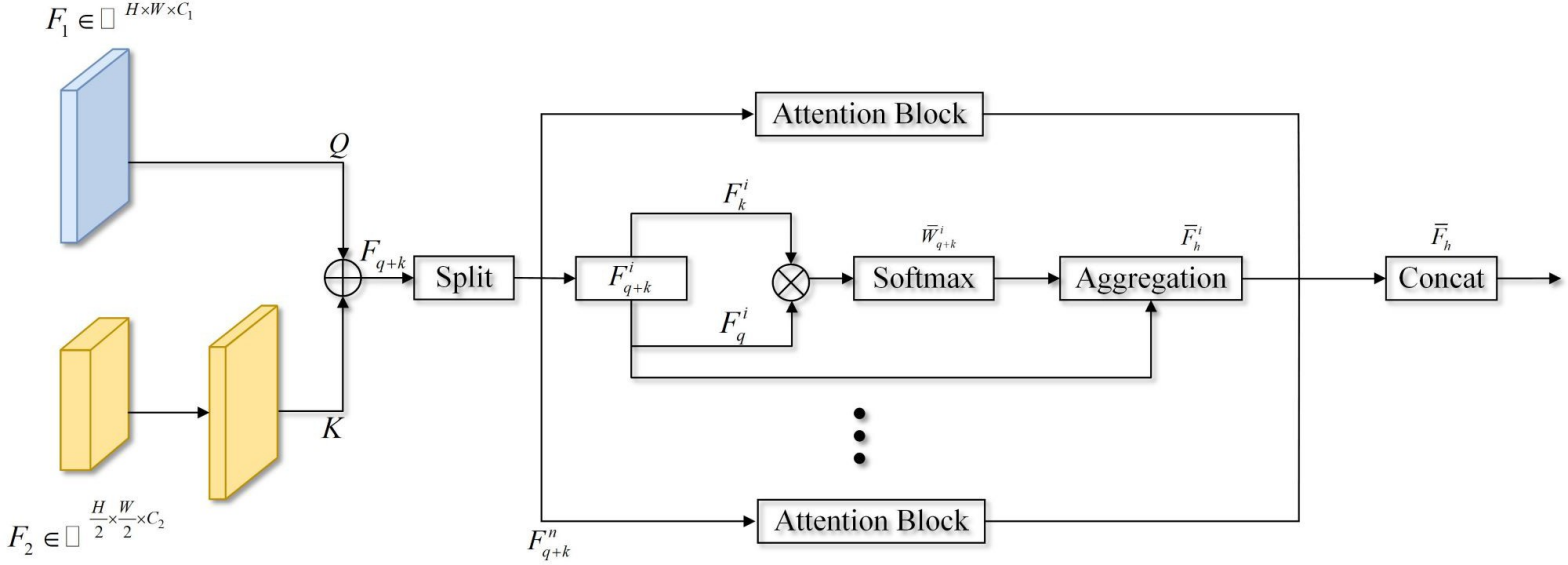
The structure of the attention-aware aggregation module (APAM).

The operation process of the attention-aware aggregation module is shown in Fig 4 In the multibranch network structure, the high-resolution branch can preserve the rich spatial detail information of the input image. The low-resolution branch captures the high-level semantic information of the image through continuous downsampling, which results in the loss of considerable low-level detail information. The feature fusion methods that are commonly used in image segmentation tasks pay more attention to the global semantic relationships between features, which leads to the problem of discontinuous details during the process of fusing different feature levels. To overcome this problem, this paper proposes an APAM structure.

The APAM module first converts feature *F*_*1*_ into Q, upsamples feature *F*_*2*_ to ${\mathbb{R}}^{H\times W\times C_{1}}$, then converts it into *K*, and finally splices *Q* and *K* to obtain a set of hierarchical features *F*_*q+k*_ through the splicing operation Concat(∙). This set of features is divided into N blocks according to the channel dimension, and Attention(∙) is performed on each block in turn. $F_{q+k}^{i}$ is used to calculate the attention weight $W_{q+k}^{i}$ of block *i*, as shown in Eq (7). Then, the attention weight $W_{q+k}^{i}$ is subjected to the Softmax(∙) normalization operation and weighted with $F_{q+k}^{i}$ to obtain an attention-enhanced hierarchical representation ${\bar{F}}_{h}^{i}$ of the ith block, as shown in Eq (8). Finally, the attention-enhanced hierarchical representation of N blocks is spliced along the channel dimension to obtain the output ${\bar{F}}_{h}$ of the APAM module, as shown in Eq (9). The APAM module considers both local details and global semantics and fully integrates high-resolution spatial detail features and low-resolution contextual semantic features.


$$W_{q+k}^{i}=F_{q}^{i}\cdot F_{k}^{i}$$ (7)

$${\bar{F}}_{h}^{i}=\mathrm{Attention}(F_{q+k}^{i})=\sum_{l}^{L}\mathrm{softmax}(W_{q+k}^{i})\cdot F_{q+k}^{i}$$ (8)

$${\bar{F}}_{h}=\mathrm{Concat}({\bar{F}}_{h}^{1},{\bar{F}}_{h}^{2},\cdots ,{\bar{F}}_{h}^{n})$$ (9)

#### Category awareness module

In semantic segmentation tasks, different categories of objects have unique appearance, shape and texture features. Traditional semantic segmentation networks typically use shared feature representations to process all categories, which ignores the feature differences between different categories. Therefore, the feature representations of some categories may not be sufficiently fine or effective, which affects the resulting segmentation performance. In addition, the classification decision obtained for each pixel depends not only on the characteristics of the pixel itself but also on the surrounding pixels and the global context. Traditional semantic segmentation networks typically use the convolution operations of local receptive fields to process the features of pixels, which leads to their inability to fully utilize global context information.

**Fig 5:**
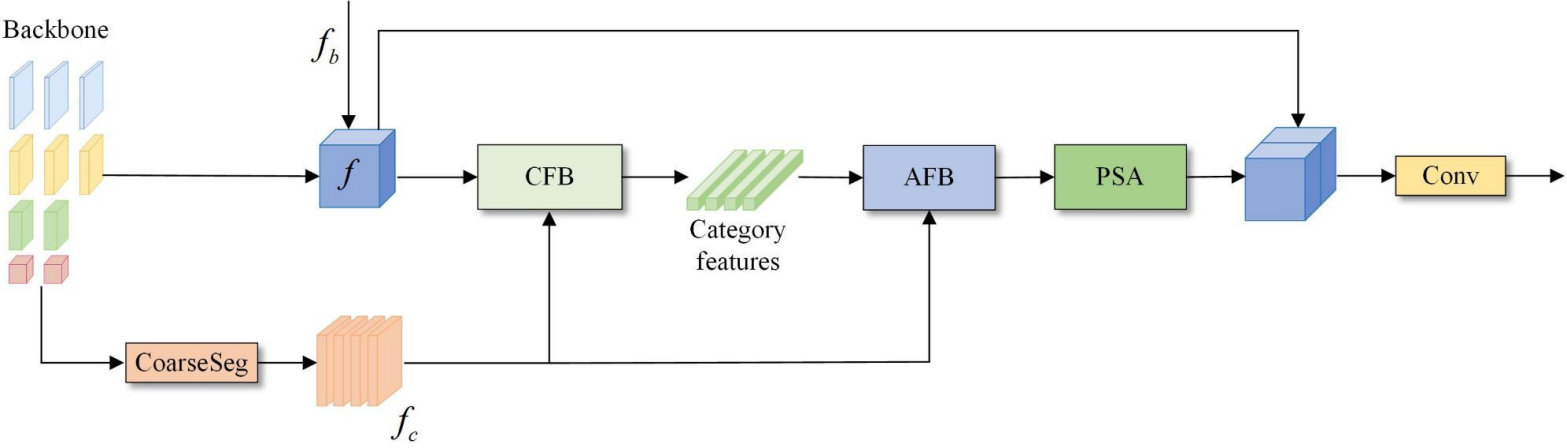
The structure of the category awareness module (CAM). PSA, enhanced polarization attention; AFB, attention feature block; CFB, category feature block.

**Fig 6:**
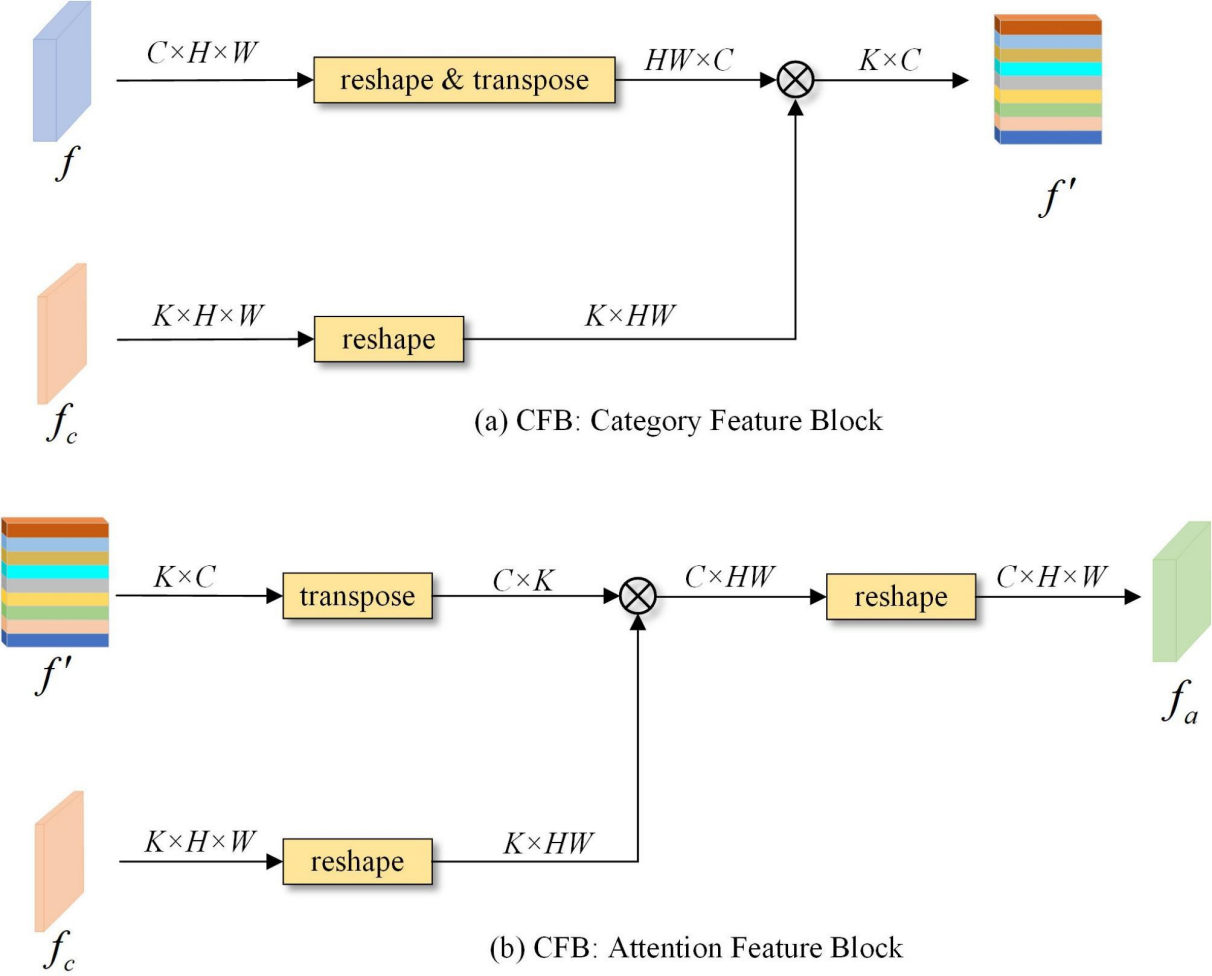
The structural diagrams of the category feature block (a) and attention feature block (b).

**Fig 7:**
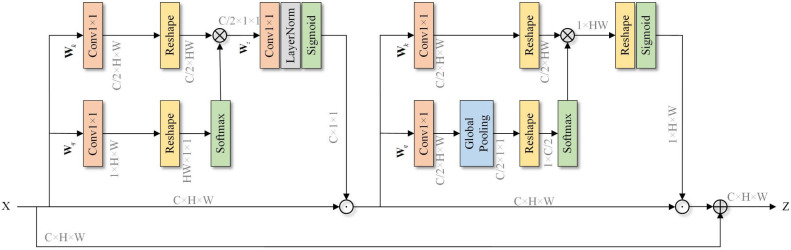
Structure diagram of the improved enhanced polarization attention (PSA) module.

In view of the above two problems, the ECMNet model proposed in this paper explicitly learns the feature representation of each category by adding a category awareness module (CAM) to capture the feature differences between categories. The overall structure of the category awareness module is shown in Fig 5. First, a coarse segmentation result *f*_*c*_ is estimated according to the feature representation of the backbone network, and each channel corresponds to a category segmentation result. Then, the deep feature representation *f* is utilized with the category feature block (CFB) operation to determine the category feature representation. The CFB structure is shown in Fig 6(A). First, the dimensionality of the coarse segmentation graph $f_{c}\in {\mathbb{R}}^{K\times H\times W}$ is reshaped to ${\mathbb{R}}^{K\times HW}$ via the reshaping operation, and then the dimensionality of the deep feature $f\in {\mathbb{R}}^{C\times H\times W}$ is reshaped and transposed to obtain ${\mathbb{R}}^{HW\times C}$, which is calculated via matrix multiplication to obtain *K* sets of vectors. Each vector corresponds to a feature representation of a semantic category. After that, the class feature representation $f^{\prime }\in {\mathbb{R}}^{K\times C}$ and the coarse segmentation image are employed by the attention feature block (AFB) to obtain the class attention feature representation $f_{a}\in {\mathbb{R}}^{C\times H\times W}$ so that different pixels can selectively focus on different categories. The AFB structure is shown in Fig 6(B). Finally, an enhanced polarization attention module (PSA) is introduced after the class attention feature, which aims to enhance the ability of the model to express the category target by establishing remote dependency relationships between pixels. The polarization attention module contains two branches in the channel and spatial dimensions. By fully folding the features in one direction and maintaining orthogonality in the other direction to reduce the information loss, the Softmax function is used to enhance the attention range and perform dynamic mapping on the minimum tensor. To ensure the appropriate number of calculations, the module can effectively perform long-distance modeling while connecting the attention branches in series and adding residual connections to retain some features for avoiding the information loss caused by the polarization self-attention mechanism. The whole process is shown in Fig 7.

### Edge sensing optimization and deep supervision strategy

**Fig 8:**
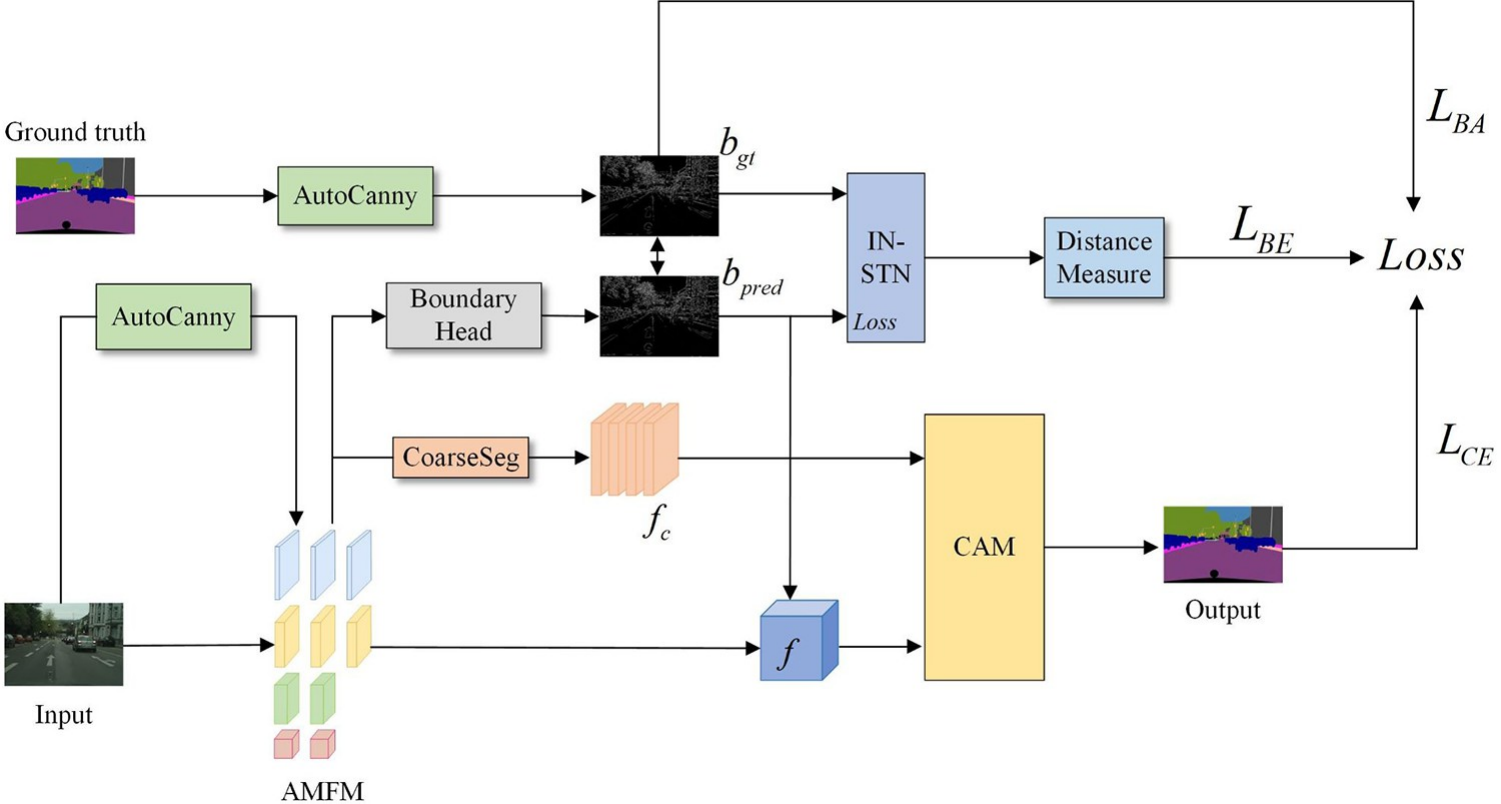
Deep training structure diagram of the ECMNet model.

With the deepening of convolution and pooling operations in deep neural networks, boundary detail information is lost, which in turn affects the classification results obtained target boundary pixels. Therefore, this paper integrates edge information into the network to provide useful prior knowledge so that the network can pay more attention to the boundary of the object. Moreover, a joint loss function based on edge perception optimization is proposed to strengthen the attention paid to object edges during the training process. To solve the fuzzy boundary problem of networks in object segmentation tasks, finer and more accurate segmentation results are generated. The overall structure is shown in Fig 8.

The traditional Canny algorithm smooths the target gray image via Gaussian filtering and performs nonmaximum suppression on the gradient amplitude. Then, the double threshold method is used to segment the image edge information, the strong and weak edges are distinguished, and isolated weak edges are eliminated to obtain the final edge contour. However, the edge strengths and noise levels of different images may be significantly different, which leads to a decrease in the sensitivity of the edge detector to gradient changes when addressing edges with small gradient changes. Therefore, this paper proposes an improved adaptive Canny algorithm, which uses adaptive median filtering instead of Gaussian filtering for image denoising and employs the Otsu algorithm to automatically determine the appropriate threshold. This method can better adapt to local changes in an image, effectively retaining important edges while suppressing noise and weak edges. Adaptive median filtering can dynamically adjust the filter window size according to the specific input image to remove noise while retaining image detail features. The operation steps are as follows. Assume that *M*_max_, *M*_min_ and *M*_med_ are the maximum, minimum and middle gray values in the action area of the filter, respectively, and that *M*_(x,y)_ is the gray value of image (x,y). Process 1: If *M*_med_−*M*_min_>0 and *M*_med_−*M*_max_<0, process 2 is executed; otherwise, the filter action area is expanded, and process 1 is re-executed. If the expanded filter action area exceeds the maximum window size, then *M*_*med*_ is output. Process 2: If *M*_(x,y)_−*M*_min_>0 and *M*_(x,y)_−*M*_max_<0, *M*_*(x*,*y)*_ is output; otherwise, *M*_*med*_ is output. The high- and low-threshold selection processes of the adaptive Canny algorithm are as follows:

lowTH=max(0,median*0.66) (10)

highTH=min(median*1.33,255) (11)

where *highTH* represents a high threshold, *lowTH* represents a low threshold, *median* is the median of all pixel values in an image, *max* is the maximum operation for an interval, and *min* is the minimum operation for an interval. The coefficient selection process refers to the settings of previous researchers, and the selected coefficient makes the extraction effect of the edge contour relatively good.

In this paper, the improved adaptive Canny algorithm is used to obtain edge information, which is applied to the multibranch feature extraction backbone network and the context feature segmentation head module. The specific steps are as follows. First, edge detection is performed on the input image and the coarse segmentation image to obtain edge information *X*_*1*_ and *X*_*2*_, respectively. Then, global average pooling and 1×1 convolution operations are performed on this edge information, and this step is activated by the sigmoid activation function. Finally, the processed edge information is fused with the features of each branch and the segmentation head.

The loss function that is most commonly used in semantic segmentation tasks is the pixel-level cross-entropy loss. This type of loss measures only the pixel transformation between a predicted value and the corresponding real value and considers the classification accuracy at the pixel level. The attention paid by this loss to the edge is not sufficient for implementing boundary segmentation, so the segmentation results generated by the model have problems such as blurring and sawtooth effects at the boundary, which affects the quality and accuracy of segmentation. In this work, the boundary segmentation head is used to model the semantic features extracted by the multibranch backbone network as a binary image segmentation task. During the training process, the expression of shallow features on details and boundaries is strengthened, and the boundary feature information *b*_*pred*_ is integrated into feature *f*. At the same time, the improved adaptive Canny algorithm is used to extract the edge from the real segmentation map, and the real boundary segmentation map *b*_*gt*_ is generated. Then, the real boundary segmentation graph *b*_*gt*_ and the boundary segmentation graph *b*_*pred*_ predicted by the backbone network are used as the inputs of the auxiliary loss *L*_*BA*_, and the binary cross-entropy loss and Dice loss are used to increase the accuracy of boundary segmentation. The process of calculating the auxiliary loss is shown in Eq (12):

$$L_{BA}(b_{pred},b_{gt})=L_{bce}(b_{pred},b_{gt})+L_{dice}(b_{pred},b_{gt})$$ (12)

where *L*_*bce*_ is the binary cross-entropy loss and *L*_*dice*_ is the Dice loss.

Inspired by InverseForm, ECMNet introduces an inverse transformation network to learn the spatial relationship between two boundary maps in the training phase and predicts a homography change as its output. Then, the Euclidean distance measure is used to calculate the spatial distance between the boundary maps and is employed as the input of the joint loss function to supervise the training procedure of the network. This process is shown in Eq (13):

$$L_{BE}\left(b_{pred},b_{gt}\right)=\sum_{j=1}^{n}d_{if}(b_{pred,j},b_{gt,j})$$ (13)

$$d_{if}(x,t_{\theta }(x))={\left\|\hat{\theta }-I_{3}\right\|}_{F}$$ (14)

where *d*_*if*_(∙) is the reasoning process involving the Euclidean distance measure, *x* and *t*_*θ*_(*x*) are the inputs of the inverse transformation network, $\hat{\theta }$ is the output of the inverse transformation network, that is, the mapping relationship between the real and predicted boundary graphs, *I*_*3*_ is a 3×3 unit matrix, and ‖⋅‖_*F*_ is the Frobenius norm.

In summary, the process of using the joint loss function to train the network is as follows. First, the edges are extracted from the real segmentation map and the high-resolution detail feature map of the multibranch feature backbone network, and the detail prediction procedure is modeled as a binary image segmentation task by using the edge segmentation head. The joint calculation of the binary cross-entropy loss and Dice loss of the real boundary label map and the boundary map of the high-resolution branch features is used as an auxiliary loss, as shown in Eq (12). Second, the above two segmentation boundary maps are cut and fed into the trained inverse transformation network as inputs. By comparing the transformation matrix and the unit matrix of the network output, the spatial distance between the boundary maps is calculated and used as the input of the joint loss to further train the network, as shown in Eq (13).

The calculation process of the entire loss function is as follows:

$$Loss=L_{CE}+\alpha L_{BA}+\beta L_{BE}$$ (15)

where *L*_*CE*_ is the pixel-level cross-entropy loss term, *L*_*BA*_ is the auxiliary loss term, *L*_*BE*_ is the boundary loss term, and α and β are the parameters of the auxiliary loss term and the boundary loss term, respectively.

### Experiment

#### Experimental dataset

This paper utilizes two commonly used semantic segmentation datasets: Cityspaces and CamVid. The Cityspaces dataset focuses on urban street scenes from the perspectives of vehicles and records street-view photos of different scenes and seasons in different cities. It contains 5000 finely labeled pixel-level images, 2000 roughly labeled images and 30 types of labeled objects. Among them, 19 types are used for semantic segmentation tasks, and each image is an RGB-channel color image with a resolution of 2048×1024. In this work, 2975 training set images are used for training, and 500 verification set images are used for verification.

The CamVid dataset is a benchmark dataset created by researchers at the University of Cambridge for semantic segmentation tasks. It is a road and driving scene image segmentation dataset extracted from video in a frame-by-frame manner. The CamVid dataset contains 701 finely annotated image frames and provides 32 different semantic categories. The experiment in this paper uses a subset consisting of 11 semantic categories, including 367 images for model training, 233 images for testing, and 101 images for verification. The resolution of each image is 960×720.

#### Experimental setup and evaluation indices

The experimental environment of this paper is a Ubuntu system with Python 3.8, which is based on the PyTorch_1.10.0 framework. The training processes implemented by the model on the Cityspaces dataset and CamVid dataset are completed on an NVIDIA GeForce GTX 2080 Ti graphics card. In this work, we use transfer learning to pretrain the multibranch backbone network on the ImageNet dataset and load the weight parameters obtained through training to accelerate the convergence and learning processes of the model. Moreover, the image feature extraction performance of the multibranch backbone network is compared and verified.

The dataset training configurations are shown in Table 1. For the Cityspaces dataset, the model iterates for 600 epochs, the batch size is set to 8, and the input image is randomly cropped to a resolution of 1024×512 during the training phase. In the experiment, the stochastic gradient descent (SGD) method with momentum is used, and the learning rate is dynamically adjusted during training, as shown in Eq 16. For the CamVid dataset, the model iterates for 800 epochs, the batch size is set to 16, the initial learning rate is set to 0.001, the weight attenuation rate is 0.0001, and the remaining training details are the same as those of the Cityspaces dataset.

$$lr=base\_lr\times {\left(1-\frac{epoch}{max\_epoch}\right)}^{power}$$ (16)

where *lr* is the new learning rate, *base_lr* is the benchmark learning rate with its value set to 0.01, “epoch” is the number of iterations, max_epoch is the maximum number of iterations, and “power” is the factor that adjusts the change rate.

**Table 1:** Training configurations of the utilized datasets.

Various configurations	Cityspaces	CamVid
Dataset size	1024×512	960×720
Batch size	8	16
Optimizer	SGD	SGD
Learning rate	0.01	0.001
Number of iterations	600	800

In this work, the mean intersection over union (MIoU) and pixel accuracy (PA) are used as evaluation indices to objectively measure the experimental results. The intersection over union (IoU) is the intersection of the real value and the predicted value of a pixel divided by the union of the real value and the predicted value of the pixel, whereas the MIoU is the intersection over union (IoU) of the real label and the result predicted for each class; then, the mean IoU across all categories is calculated. The MIoU is a standard accuracy measurement method that reflects the degree of coincidence between a model segmentation result and the true value of the original image. Pixel accuracy is the ratio of the number of correct pixels to the total number of pixels. The IoU, MIoU, and MPA are defined as follows in Eqs (17)–(19), respectively:

$$IoU=\frac{P_{ii}}{\sum_{j=0}^{k}P_{ij}-\sum_{j=0}^{k}P_{ji}-P_{ii}}$$ (17)

$$MIoU=\frac{\sum_{i=0}^{k}IoU}{k+1}$$ (18)

$$PA=\sum_{i=0}^{k}\frac{P_{ij}}{\sum_{j=0}^{k}P_{ij}}$$ (19)

where *k+1* denotes that a total of *k+1* categories, including the background, are involved in the segmentation task, *P*_*ij*_ represents the number of pixels belonging to category *i* that are predicted as belonging to category *j*, *P*_*ji*_ represents the number of pixels belonging to category *j* that are predicted as belonging to category *i*, and *P*_*ii*_ represents the number of pixels belonging to category *i* that are predicted as belonging to category *i*.

## Results and discussion

### Validation on the Cityspaces dataset

The method proposed in this paper is verified on the Cityspaces dataset (S1 File). The model experiment is deployed according to the settings described in the Experimental setup and evaluation indices section, and the proposed approach is compared and analyzed with other advanced models.

The experimental results are shown in Table 2. Compared with other advanced methods on the Cityspaces dataset, the proposed ECMNet method achieves MIoU and PA values of 79.2% and 96.2%, respectively, with a parameter count of 35.6×10^6^. Compared with the classic DeepLabV3+ semantic segmentation method, the number of parameters in ECMNet is only ½ the amount, but the MIoU value is increased by 0.6%. Compared with the parallel HRNet multibranch semantic segmentation network, the segmentation effect is significantly improved by the proposed method, and its MIoU value is increased by 3.9%. Moreover, the multibranch network based on edge optimization and category awareness proposed in this paper strikes a better balance between model size and segmentation accuracy than do the existing multibranch method (ICNet) [33] and the STDC method based on boundary optimization. Compared with that of ICNet, the MIoU increases by 8%, and compared with that of the STDC method, the MIoU increases by 2.2%.

**Table 2:** Experimental comparison among different algorithms on the Cityspaces validation set.

Model	Backbone	MIoU/%	PA/%	Params/10^6^
PSPNet[16]	ResNet-50	76.7	94.8	46.8
PSPNet[16]	ResNet-101	78.5	95.6	65.7
DeepLabV3+[18]	ResNet-101	78.6	95.5	75.8
BiseNet[34]	ResNet-101	74.8	94.3	55.2
ICNet[33]	PSPNet-50	71.2	93.1	28.5
OCNet[35]	ResNet-101	73.1	93.4	95.6
ENCNet[10]	ResNet-50	72.3	93.2	36.2
PSANet[36]	ResNet-101	76.5	94.7	70.5
DenseASPP[13]	DenseNet-169	71.9	93.5	28.6
HRNet[25]	HRNetV2p-W48	75.3	94.1	65.9
STDC[32]		77.0	95.3	25.2
ECMNet (Ours)	AMFM	79.2	96.2	35.6

In this work, the MIoU values of each type of object are compared on the verification set of the Cityspaces dataset. The classic HRNet parallel multibranch network, the classic OCNet context semantic segmentation network and the edge-enhanced STDC approach are selected as the comparison models and compared with the ECMNet method proposed in this paper. The comparison results are shown in Table 3, and all the values in the table are percentiles. The results show that the MIoU values yielded by the proposed method for roads, sidewalks, buildings, walls, fences, poles, lights, signs, vegetation, the sky, cars, trucks, buses, trains, motorcycles, and bicycles are 98.6%, 86.5%, 93.6%, 59.7%, 63.2%, 64.7%, 70.6%, 77.7%, 93.4%, 95.8%, 95.5%, 84.9%, 90.6%, 81.9%, 63.1%, and 76.8%, respectively. ECMNet adds a multibranch bidirectional detail enhancement structure and an attention-aware fusion structure to the feature extraction process, which makes the segmentation results obtained for slender small targets such as fences and poles more accurate. Because the category awareness module extracts effective context information and category feature information, this method also has a good segmentation effect on large targets such as trucks, buses, trains, and buildings. Owing to the optimization strategy of the edge perception optimization module and the joint loss function of the fusion edge, the method in this paper segments more complex types of edges, such as buildings, indicator lights, plants, and public transportation, in more detail.

**Table 3:** Comparison among various experimental results produced on the Cityspaces validation set.

class	HRNet	OCNet	STDC	ECMNet
road	98.1	97.7	98	98.6
sidewalk	83.6	82.8	84.3	86.5
building	92.0	90.7	92.0	93.6
wall	46.1	47.2	56.7	59.7
fence	52.5	49.9	60.1	63.2
pole	63.0	55.6	62.6	64.7
t-light	70.3	66.4	68.6	70.6
t-sign	75.6	70.8	76.5	77.7
vegetation	92.9	91.7	92.2	93.4
terrain	70.1	69.8	66.7	65.9
sky	95.2	94.2	94.4	95.8
person	83.8	80.3	80.7	81.7
rider	64.3	62.7	62.0	60.5
car	95.0	94.0	94.7	95.5
truck	63.6	64.0	80.2	84.9
bus	77.7	75.5	85.6	90.6
train	71.7	63.7	78.7	81.9
motor	61.3	61.7	53.7	63.1
bike	73.4	69.4	74.5	76.8
MIoU	75.3	73.1	77.0	79.2

**Fig 9:**
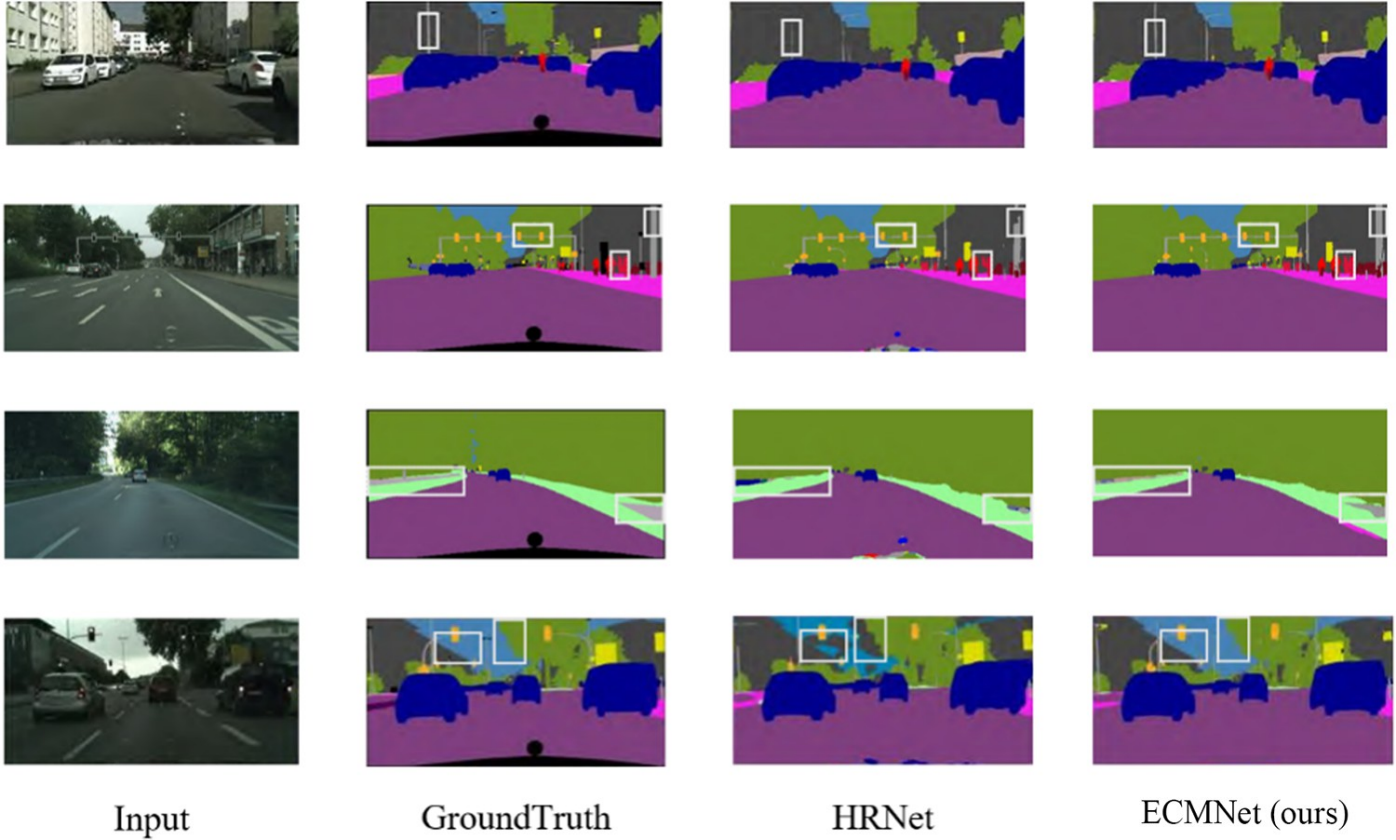
Visualization comparison results obtained on the Cityspaces dataset.

To intuitively show the segmentation effect of the multibranch segmentation network on the Cityspaces dataset, the HRNet+OCR model with high- and low-resolution multibranch structures and a context semantic enhancement module similar to that used in this paper is selected as the comparison model for verification purposes, the segmentation results predicted for the verification set picture are visually compared, and parts of them are selected for display. The effect is shown in Fig 9. Fig 9 demonstrates that, compared with the HRNet+OCR method, the proposed method is more effective at segmenting fine rods, fences, signal lights and other categories, and its segmentation details are obvious. The fourth line comparison diagram shows that the comparison method incorrectly identifies some sky areas and trees as buildings. The method in this paper more accurately segments buildings and trees. In the complex case with dim light and close spacings between objects, the method developed in this paper can better identify and segment areas such as buildings, the sky and trees. This is due to the addition of the edge optimization module, which focuses on the fusion of edge feature information, and the deep supervision of the joint loss function containing the boundary loss term of the network.

### Validation on the CamVid dataset

The method proposed in this paper is verified on the CamVid dataset, and the model experiment is deployed according to the settings described in the Experimental setup and evaluation indices section and compared with other advanced models. In the training stage and the reasoning stage, the input image resolution of the experimental comparison models is 970×720.

The experimental results are shown in Table 4. The segmentation index (MIoU) achieved by the proposed method on the CamVid dataset reaches 79.6%. Compared with those of the multibranch image segmentation methods (HRNet and ICNet), the MIoU is increased by 3.5% and 10.3%, respectively, when the number of parameters is only half that HRNet and not much improved compared with that of ICNet, which indicates that the attention-guided multibranch fusion structure (AMFM) better retains spatial detail information and edge feature information. Compared with that of OCNet possessing a contextual semantic feature module, the MioU of the proposed model is improved by 7.4%; that is, the category awareness module utilized in this paper has a better effect on the classification decision of each pixel. Compared with the STDC method with edge enhancement processing, although the number of parameters is increased, the MIoU is increased by 3.0%. The algorithm in this paper achieves a better balance between its model size and segmentation effect.

**Table 4:** Experimental comparison among different methods on the CamVid dataset.

Model	Backbone	MIoU/%	Params/10^6^
PSPNet [16]	ResNet-50	74.4	46.6
PSPNet [16]	ResNet-101	77.3	65.8
DeeplabV3+ [18]	ResNet-101	77.8	75.5
BiseNet [34]	ResNet-101	70.7	56.1
ICNet[33]	PSPNet-50	69.3	27.9
OCNet [35]	ResNet-101	72.2	95.4
ENCNet [10]	ResNet-50	71.5	36.7
PSANet[36]	ResNet-101	76.0	70.6
DenseASPP [13]	DenseNet-169	68.6	28.9
HRNet [25]	HRNetV2p-W48	76.1	65.1
STDC [32]		76.6	25.8
ECMNet (Ours)	AMFM	79.6	35.6

### Ablation experiment

In this paper, a series of ablation experiments are conducted on the Cityspaces and CamVid datasets to verify the modules of the proposed method. The effectiveness of each module is verified on the validation set and test set of the Cityspaces dataset, and the MIoU and PA are used as indicators to measure the segmentation effect. As shown in Table 5, the AMFM is the attention-guided multibranch fusion backbone network proposed in this paper. At the single scale of the validation set, the MIoU value of the AMFM can reach 77.1%, and the PA value can reach 94.7%. Under the single-scale setting of the test set, the MIoU value reaches 76.7%, and the PA value reaches 94.6%. The CAM is added to the backbone network to capture the feature differences between different categories. By guiding the categories of the pixels, the classification accuracy achieved for these pixels is improved, and the overall segmentation effect is also improved. The MIoU value is increased by 0.9% on the verification set and by 1.1% on the test set, and the PA value is increased by 1.2% on both the verification set and the test set. On this basis, the EOM is added to this method, and the joint loss function is used for training to enhance the edge detail information contained in the network features, improve the attention paid to object edges, and improve the edge segmentation effect. The MIoU and PA values reach 79.2% and 96.2%, respectively, on the verification set and 96.2% and 96.0%, respectively, on the test set. In summary, from the perspective of the segmentation effect, each module proposed in this paper has a good effect on semantic image segmentation.

**Table 5:** Performance comparison among the proposed modules on the Cityspaces dataset.

Module	MIoU		PA	
	Val	Test	Val	Test
AMFM	77.1	76.7	94.7	94.6
+CAM	78.0	77.8	95.9	95.8
+EOM	79.2	78.8	96.2	96.0

Moreover, this paper also verifies the effectiveness of each module on the CamVid dataset and uses the MIoU and PA indicators to measure the impact of each module on the overall segmentation effect. The results are shown in Table 6. The MIoU value and PA value of the attention-guided multibranch fusion backbone network proposed in this paper reach 77.6% and 95.2%, respectively. The CAM is added to the AMFM to optimize the overall classification decision for each pixel, and the MIoU value and PA value increase by 0.7% and 1.1%, respectively. Finally, the EOM is added, and the training process is optimized. The MIoU and PA values of the ECMNet model developed in this paper reach 79.6% and 96.8%, respectively. The experiments show that the modules proposed in this paper yield good results in semantic image segmentation tasks.

**Table 6:** Performance comparison among the proposed modules on the CamVid dataset.

Module	MIoU	PA
AMFM	77.6	95.2
+CAM	78.3	96.3
+EOM	79.6	96.8

To better verify the effectiveness of the attention-guided multibranch fusion backbone network proposed in this paper, the AMFM backbone network and the commonly used feature extraction network are compared in terms of their classification accuracy on the ImageNet-1k dataset to verify the feature extraction ability of the developed approach. The number of model parameters can reflect the structure of a model and affect the memory or video memory required for model operations. The obtained experimental results are shown in Table 7. The top-1 classification accuracy of the proposed method on the ImageNet-1k dataset reaches 77.5%, which is similar to that of ResNet-101, but the number of parameters is only 1/3 of this approach. The proposed method achieves a better balance between classification accuracy and method scale.

**Table 7:** Comparison among common backbone networks on the ImageNet-1k dataset.

Backbone	Top-1/%	Params/10^6^
ResNet-50 [37]	76.2	25.6
ResNet-101[37]	77.4	44.5
VGG-16 [38]	74.4	138
HRNet-W48 [25]	78.5	63.6
AMFM (Ours)	77.5	16.5

In addition, to balance the total loss, auxiliary loss and boundary loss during the training process, this paper also performs experiments on the Cityspaces dataset to determine the weights of the auxiliary loss and boundary loss items. The experimental results are shown in Tables 8 and 9, respectively. In this work, the weight α of a single loss term is verified first. When the value of α is 0.25, the experimental segmentation effect is the best, and the MIoU value reaches 78.5%. Second, the weight β of the loss term is verified, and the value of α remains constant at 0.25. The experimental results show that when the value of β is 0.5, the effect is optimized, and the MIoU value reaches 79.2%.

**Table 8:** Results of the value experiment concerning the auxiliary loss weight α.

α	MIoU (α)
0.1	78.1
0.25	78.5
0.5	78.3
0.75	78.2

**Table 9:** Results of the value experiment concerning the boundary loss weight β.

β	MioU (β)
0.1	78.7
0.25	79.0
0.5	79.2
0.75	78.9

## Conclusion

To address the object edge blurring and confusion problems encountered during the semantic image segmentation process, this paper proposes a multibranch semantic segmentation method called ECMNet, which is based on boundary optimization and category perception. First, an attention-guided multibranch fusion backbone network is proposed. The depth and computational complexity of the network are reduced by the use of a parallel multibranch structure. An improved adaptive Canny algorithm is used to extract edge contours. For the preprocessed features, a bidirectional detail enhancement module is used to enhance the feature information interactions between branches with different resolutions, which effectively integrates high-resolution detail feature information and low-resolution contextual semantic information. Second, a category-aware module is added to learn the feature representation of each category, and the feature differences between categories are captured. Global context information is introduced, and category feature information is integrated into the feature representations of the pixels to provide context-dependent guidance. Finally, an edge optimization module and a depth supervision strategy for the joint loss function are proposed. The adaptive median filter is used to replace the Gaussian filter to denoise the input image, and the Otsu algorithm is used to adaptively determine the appropriate threshold. The segmentation effect of the model on the edge of an object is optimized without affecting the reasoning speed of the model. The experimental results show that on the Cityspaces dataset, compared with those of the classic parallel multibranch semantic segmentation network (HRNet), the MIoU value is increased by approximately 3.9%, and the PA value is increased by approximately 2.1%. On the CamVid dataset, compared with that of HRNet, the MIoU value is increased by 3.5%, which shows that this method can achieve better segmentation results for most objects contained in the input image. Compared with those of the DenseASPP method, which has fewer parameters and a better segmentation effect, the parameter quantity of the proposed method increases by approximately 3×10^6^, whereas the MIOU increases by 7.3%. Therefore, the method developed in this paper is more balanced in terms of its parameter quantity and segmentation effect. Moreover, when the MIoU values produced for each type of object in the Cityspaces dataset are compared, the method in this paper more accurately segments slender small targets such as fences and poles and has a stronger recognition ability for objects with close intervals.

However, in the experimental results obtained on the Cityspaces dataset, the proposed method has a poor segmentation effect in the face of targets with relatively complex postures and richer details, such as pedestrians, cyclists and other categories. In addition, we did not perform comparison between the model proposed in this study and those reported in the literature in terms of FLOPS efficiency, and the model proposed here might have certain limitations in terms of this metric due to the large number of parameters involved. In the future, the feature extraction ability of the proposed approach for small-scale targets in images will be enhanced by optimizing the loss function and improving the feature extraction model for such problems to better meet the application demands in complex scenarios.

## Supporting information

S1 File Code and raw data used in this study. (RAR)
